# Clinical factors affecting depression in patients with painful temporomandibular disorders during the COVID-19 pandemic

**DOI:** 10.1038/s41598-022-18745-0

**Published:** 2022-08-29

**Authors:** Yeon-Hee Lee, Q-Schick Auh

**Affiliations:** Department of Orofacial Pain and Oral Medicine Kyung Hee University Dental Hospital, Kyung Hee University, #613 Hoegi-dong, Dongdaemun-gu, Seoul, 02447 South Korea

**Keywords:** Psychology, Diseases, Health care, Medical research, Risk factors, Signs and symptoms

## Abstract

Temporomandibular disorders (TMD) are a multifactorial condition associated with both physical and psychological factors. Stress has been known to trigger or worsens TMD. We aimed to investigate whether the novel coronavirus disease-2019 (COVID-19) pandemic aggravates depression in patients with painful TMD, and the factors that affect their level of depression. We included 112 patients with painful TMD (74 females, 38 males; mean age: 35.90 ± 17.60 years; myalgia [n = 38], arthralgia [n = 43], mixed joint–muscle TMD pain [n = 31]). TMD was diagnosed based on the Diagnostic Criteria for TMD Axis I. Physical pain intensity was recorded using the visual analog scale (VAS); psycho-emotional status (depression: Beck Depression Inventory [BDI], anxiety: Beck Anxiety Inventory [BAI], and generalized stress related to COVID19: Global Assessment of Recent Stress [GARS]) was investigated twice (before [BC] and after COVID-19 [AC]). Additionally, factors affecting BDI-AC were investigated. BDI (p < 0.001), BAI (p < 0.001), GARS (p < 0.001), and VAS (p < 0.01) scores were significantly increased at AC than BC. The depression, anxiety, and stress levels were significantly positively correlated, and the AC and BC values of each factor showed a high correlation. In the mixed TMD group, BDI-AC was positively correlated with VAS-AC (p < 0.001). In the multiple regression analysis, clenching habit was the strongest predictor of an increase in the BDI scores from moderate to severe, followed by psychological distress, muscle stiffness, female sex, BAI-AC, and TMJ sounds. COVID-19 has negatively affected the psycho-emotional state of patients with painful TMD, and several clinical factors, including female sex and clenching habits, have influenced depression.

## Introduction

In December 2019, a cluster of pneumonia cases caused by SARS-CoV-2 was reported in Hubei, China^1^. Subsequently, the illness caused by this virus was termed coronavirus disease-2019 (COVID-19). As of February 2022, SARS-CoV-2 and SARS-CoV-2 variants have killed 5.87 million people worldwide, and it continues to take a toll on people’s health. During the pandemic, various socio-economic issues, physical restrictions, restrictions on outdoor activities in sunlight, psychological withdrawal, and limited access to medical care have worsened the living conditions^2^. COVID-19 is a significant modern-day medical challenge, even in the clinical field of temporomandibular disorders (TMD), which is the most common cause for orofacial pain of non-odontogenic origin.

Psychological diseases, including depression and anxiety, is a major global health-related burden. Approximately 16.6% of individuals experience depression at some point in their life^3^. COVID-19 has been a major cause for stress. Although depressive and anxiety disorders have been leading contributors to the health-related burden during the pandemic^4^, its global impact remains largely unknown. However, a significant increase of > 25% in the prevalence of both major depressive and anxiety disorders has been observed since the beginning of the pandemic^5^. Additionally, an increased prevalence of depression and anxiety has been observed in both males and females across all ages in 204 countries^6^. Psychological distress and unstable and weakened psycho-emotional status are known to be associated with increased intensity and duration of pain. Therefore, with the progression of COVID-19, the need to understand and respond to TMD pain with long COVID is increasingly pressing.

TMD is an umbrella term for a group of musculoskeletal and neuromuscular conditions that involve the temporomandibular joint (TMJ), masticatory muscles, and adjacent structures. TMD often presents with pain in the jaw, face, and neck and/or with dysfunction of the TMJ, and is commonly accompanied by headache and/or ear pain^7^. TMD affects ~ 10–15% of the general population, with a female predominance (female:male, ~ 2:1)^8^. TMDs have multifactorial etiologies caused by both physical and psychological factors, which are expressed as Axis I and Axis II in the Diagnostic Criteria for TMD (DC/TMD), respectively^9^. Psychological stress triggers or worsens TMD signs and symptoms; correspondingly, prolonged signs and symptoms worsen psychological status^10^. Although Axis I and Axis II are not considered separate in TMD, the mechanism underlying these interrelationships is unclear. Furthermore, studies examining physical and clinical characteristics and psychological aspects, including depression in patients with TMD accompanied with COVID-19, are lacking.

COVID-19 has created an environment in which several determinants of poor mental health are exacerbated, particularly in patients with pain. Among patients living with pain, the lockdown and social isolation has been a major stressful event due to poor accessibility to treatment^11^, with the possibility of an increase in symptoms associated with COVID-19. In patients with pre-existing TMD, symptoms may be exacerbated during stressful events^12^. COVID-19 has also been associated with increased prevalence of depressive symptoms, psychological stress, and pain related to TMD^13^. In one report, more than 50% of patients with TMD reported worsening of symptoms, and that their worsening pain was associated with the stress experienced due to the COVID-19 lockdown^14^. COVID-19 has significant adverse effects on psycho-emotional status, resulting in the exacerbation of bruxism and TMD symptoms^15^. Patients with chronic TMD, in particular, are more susceptible to COVID-19 distress, with deterioration of psychological status, worsening features of central sensitization, and increased chronic facial pain severity^16^. During multimodal inpatient treatment for pain, mood deterioration and increase in pain was observed in 70% and 44% patients, respectively, and COVID-19 was associated with the chronicity of the disease^17^. Speculand et al. have provided insight into the stressful events associated with the onset and worsening of TMD symptoms^18^. However, a comprehensive approach to determine whether depression in patients with TMD is exacerbated by COVID-19 and the factors influencing it have not yet been achieved.

The physical and psycho-emotional dimensions of TMD pain and comorbid depression may be widely connected. The COVID-19 pandemic may be threaten and elevate pain and depression in people with TMD, as it does in other chronic pain patients^19^. Patients in pain should exercise isolation or social distancing since the COVID-19 pandemic could cause their physical and emotional well-being to deteriorate^20^. Neurobiological, psychological, and social factors of pain form a complex circle, thus requiring a biopsychosocial therapeutic approach. Therefore, we aim to (1) investigate the clinical and psychological characteristics of patients with painful TMD in the context of the COVID-19 pandemic, (2) analyze the relationship between depression, anxiety, psychological distress, and pain intensity in patients with TMD, and (3) investigate the psychological and clinical factors affecting depression among patients with TMD. We hypothesized that the intensity of pain or depression in patients with painful TMD worsened during the COVID-19 pandemic and that the psycho-emotional status of pre-COVID-19 patients may have an effect on current depression. A subsequent hypothesis was that the factors influencing depression may differ depending on the source of pain in patients with TMD.

## Materials and methods

### Study population

We conducted a cross-sectional survey with 112 patients (74 females [66.1%]; mean age, 35.90 ± 17.60 years) with painful TMD who visited the Department of Orofacial Pain and Oral Medicine of Kyung Hee University Dental Hospital (Seoul, South Korea). They voluntarily participated for management of painful TMD from March 2021 to October 2021. All patients were examined by two TMD specialists (L.Y-H. and A.Q-S) with > 7 years of experience in TMD diagnosis based on the DC/TMD Axis I criteria^9^.

The exclusion criteria comprised: (1) patients aged < 18 years, (2) patients with other systemic muscular disorders (e.g., fibromyalgia, rheumatoid arthritis, inflammatory joint disease), (3) patients with neurologic impairment or diseases (e.g., stroke, tumor, epilepsy), (4) pregnancy, (5) patients with a history of psychiatric disorders, and (6) inability to provide informed consent.

The inclusion criteria were diagnosis of painful TMD according to the DC/TMD Axis I classification, report of pain at the TMJ and/or masticatory muscles for > 3 months. The TMD signs and symptoms were present both before and after the declaration of the COVID-19 pandemic, and these parameters were compared. Patients completed a comprehensive questionnaire that included the DC/TMD and the Oral Behavior Checklist. Patients with painful TMD were divided into three groups as follows: pain of muscle origin (myalgia, n = 38), pain of joint origin (arthralgia, n = 43), and muscle–joint mixed TMD pain (mixed TMD pain, n = 31). The sample size of each group was above 30, so there was no difficulty in obtaining general statistical significance of the data for each group^21^.

All participants provided written consent for the study, which was approved by the Ethical Committee of Kyung Hee University Dental Hospital (KHD IRB no. KH-DT21023). This study was conducted in accordance with the principles of the Declaration of Helsinki.

### Clinical data collection

Experienced orofacial pain specialists conducted the comprehensive clinical and radiographic examinations. TMD pain and clinical disease characteristics were assessed using standard, validated, and reliable self-reported questionnaires.

#### Characteristics of TMD pain

The duration of pain derived from the TMJ and/or masticatory muscles was reported in days. TMD pain was scored subjectively by the patients, ranging from 0 (no pain at all) to 10 (worst pain imaginable) using a visual analog scale (VAS). All patients scored TMD pain with VAS at two time points: before the declaration of the COVID-19 pandemic (BC) and the present time after the WHO declaration (AC). The VAS in AC was the current value at the time point AC. VAS in BC was also obtained at time point AC assuming the TMD situation at that time.

#### Clinical factors

TMJ sounds were recorded as present when a clicking, popping, or crepitus sound was audible in the TMJ on either side. Mouth opening limitation (MOL) was defined as < 30 mm gap between the maxillary and mandibular incisal tips, and a complaint of muscle stiffness that can be confirmed clinically. TMJ locking involves locking during mouth opening or closing, and is recorded as present when one cannot open or close their mouth, respectively, at will.

#### Contributing factors and comorbidities

We investigated self-reported parafunctional activities using the Oral Behavior Checklist, which includes jaw-related behaviors such as teeth clenching and bruxism^22^. Headache was evaluated using the dichotomous question, “Do you have any headaches associated with TMD?” The presence of self-assessed tinnitus, sleep problems, psychological distress, family history, and microtrauma history were also reported with a binary answer. Each variable was recorded as a binary answer (yes/no) for all patients, as described in our previous study^10^.

### Psychological distress

The World Health Organization (WHO) declared the COVID-19 outbreak as a global pandemic on March 11, 2020^23^. In this study, three questionnaires were used to examine the psychological aspects of patients with TMD, and they completed the questionnaires at two time points: before the declaration of the COVID-19 pandemic (BC), and the present time after the WHO declaration (AC). When the patient visited the hospital (AC), questionnaires were obtained at both time points (AC and BC). At AC, the patient filled out the questionnaire, looking back at their situation, and the BC value was obtained.

#### Beck Depression Inventory-II (BDI-II)

The 2nd edition of the Beck Depression Inventory-II (BDI-II) consists of 21 items evaluated on a 4-point Likert scale (0–3) to measure the severity of depressive symptoms. The total BDI score is considered key in determining depression severity. Higher total BDI scores and levels indicate more severe depressive symptoms. The standard cut-off scores for each level were: 0–9, minimal depression; 10–18, mild depression; 19–29, moderate depression; 30–63, severe depression^24^. Total BDI scores (BDI-BC, BDI-AC) and levels (BDI level-BC, BDI level-AC) were recorded before and after the COVID-19 declaration.

#### Beck Anxiety Inventory (BAI)

The Beck Anxiety Inventory (BAI) consists of 21 self-reported items (4-point scale) and is used to assess the intensity of physical and cognitive anxiety symptoms during the past week (score range, 0–63). The standard cut-off scores for each level were: normal, 0–7; mild, 8–15; moderate, 16–25; and severe, 26–63. Among the most widely used anxiety measures, the validity and reliability of the BAI have been verified^25^. Higher total BAI scores and levels indicate more severe anxiety symptoms. The BAI total scores (BAI-BC, BAI-AC) and levels (BAI level-BC, BAI level-AC) were recorded.

#### The Global Assessment of Recent Stress (GARS) scale

The Global Assessment of Recent Stress (GARS) scale evaluates the stress perception over the past 6–24 months based on recent life changes^26^. It is based on eight sub-items on work/school-life, interpersonal relationships, relationship changes, illness and injury, economic problems, non-routine events, changes in daily life, and overall stress level. Stress was evaluated on a scale of 0–9 for each of the eight sub-items (0–72), with higher scores indicating higher stress^27^. The GARS total scores (GARS-BC, GARS-AC) were recorded and analyzed.

### Statistical analysis

The data were analyzed using SPSS Statistics for Windows, Version 26.0, (IBM Corp., Armonk, NY, USA). Continuous variables are presented as means and standard deviations (SD), and categorical variables are presented as frequencies and percentages. The inter-rater reliability between the two experts in the diagnosis of painful TMD was assessed using Cohen’s kappa coefficient and was 0.92 for myalgia, 0.95 for arthralgia, and 0.92 for mixed TMD pain groups. In case of a discrepancy in the diagnosis between the two experts, the patient was assigned to a TMD group following an in-depth discussion.

A paired t-test was performed to compare the scores for depression (BDI), anxiety (BAI), and generalized stress (GARS), and VAS scores at BC and AC. The mean difference between the TMD groups and comparison of the mean values of the continuous variables in the three TMD groups separated by TMD pain source was analyzed using analysis of variance (ANOVA) with Tukey’s post hoc test. For categorical variables, the chi-square test and Fisher’s exact test with Bonferroni adjusted post hoc analysis were used to determine the equality of proportions. Spearman’s correlation analysis was used to determine the correlations between BDI-AC, total scores of the psychological questionnaire, and TMD pain severity. Spearman’s correlation coefficients (r) ranged from − 1 to + 1, with − 1 indicating a perfectly linear negative correlation and + 1 indicating a perfectly linear positive correlation. Generalized linear models were used to identify factors significantly correlated with the BDI-AC total score. The estimated β for BDI-AC was calculated using multiple linear regression analyses after adjusting for BDI-BC. Subsequently, logistic regression analyses were performed to identify significant predictors that increased BDI level-AC in patients with painful TMD. The odds ratio for BDI level-AC was calculated using multiple logistic regression analysis after adjusting for BDI-BC. For all analyses, a two-tailed p-value < 0.05 was considered statistically significant.

### Institutional review board statement

The research protocol was reviewed in compliance with the Helsinki Declaration and approved by the Institutional Review Board of Kyung Hee University Dental Hospital (KHD IRB no. KH-DT21023).

### Informed consent statement

Informed consent was obtained from all the subjects involved in the study.

## Results

### General description

The distribution of demographics, clinical characteristics, contributing factors, and psychological distress in patients with TMD are presented in Table 1 (continuous parameters) and 2 (discontinuous parameters). The female-to-male ratio of patients with TMD was 1.95:1, indicating a female-dominant tendency. TMD is considered to be sexually dimorphic and predominantly afflicts women^28^. In this study, the three TMD subgroups consisted of myalgia (n = 38), arthralgia (n = 43), and mixed TMD pain (n = 31). The age distribution was not significantly different among the three TMD groups. The VAS scores of the myalgia, arthralgia, and mixed TMD pain groups were not significantly different between the BC and AC time points.

**Table 1:** Comparison of continuous parameters before and after COVID-19.

Continuous parameters	Myalgia (n = 38)	Arthralgia (n = 43)	Mixed TMD pain (n = 31)	p-value
	Mean ± SD	Mean ± SD	Mean ± SD	
**Clinical characteristics**				
Age (year)	37.18 ± 17.67	31.98 ± 16.32	33.65 ± 17.63	0.061
Symptom duration (day)	484.68 ± 1005.39	291.93 ± 485.09	508.10 ± 831.17	0.759
VAS_BC	2.78 ± 1.81	2.74 ± 2.77	3.00 ± 2.57	0.617
VAS_AC	4.49 ± 2.51	3.86 ± 2.14	4.52 ± 2.03	0.348
**Psychological distress**				
BDI_BC	**33.58 ± 9.67**	**28.77 ± 7.07**	**28.72 ± 7.97**	0.031*
BDI_AC	37.03 ± 10.93	32.81 ± 7.75	33.71 ± 11.78	0.159
BAI_BC	**7.82 ± 5.15**	**4.98 ± 4.47**	**4.77 ± 3.92**	0.006**
BAI_AC	**10.08 ± 7.02**	**6.44 ± 5.18**	**6.06 ± 6.01**	0.014*
GARS_BC	22.84 ± 14.60	20.70 ± 14.88	21.55 ± 12.86	0.680
GARS_AC	27.55 ± 16.84	25.05 ± 17.04	23.97 ± 16.96	0.645

The mean difference between groups was determined using ANOVA with Tukey’s post hoc test. The p-value significance was set at p < 0.05. *p-value < 0.05, **p-value < 0.01.

TMD, temporomandibular disorder; BDI, Beck Depression Inventory; BAI, Beck Anxiety Inventory; GARS, Global Assessment of Recent Stress; BC: before COVID-19, AC: after COVID-19, _BC, total score in BC; AC, total score in AC.

Significant results were bolded.

### Psychological distress before and after COVID-19

**Figure 1:**
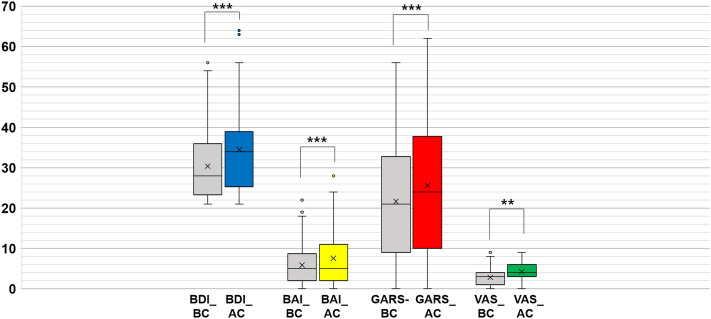
Comparison of the values of Beck Depression Inventory (BDI), Beck Anxiety Inventory (BAI), Global Assessment of Recent Stress (GARS), and Visual Analog Scale (VAS) before and after COVID-19. Results were obtained using a paired t-test. Statistical significance was set at p-value < 0.05. **p-value < 0.01, ***p-value < 0.001. TMD, temporomandibular disorder; MOL, mouth opening limitation; BDI, Beck Depression Inventory; BAI, Beck Anxiety Inventory; GARS, Global Assessment of Recent Stress; VAS, visual analog scale; BC: before COVID-19, AC: after COVID-19, -BC, total score in BC; -AC, total score in AC.

Figure 1 shows the mean differences between BC and AC for each BDI, BAI, GARS, and VAS score in 112 patients with TMD. The BDI (30.38 ± 8.52 vs. 34.49 ± 10.17, p < 0.001), BAI (5.88 ± 4.74 vs. 7.57 ± 6.29, p < 0.001), GARS (21.66 ± 14.16 vs. 25.60 ± 16.86, p < 0.001), and VAS (2.83 ± 2.40 vs. 4.28 ± 2.25, p < 0.01) scores were significantly increased at AC compared to those of BC.

**Figure 2:**
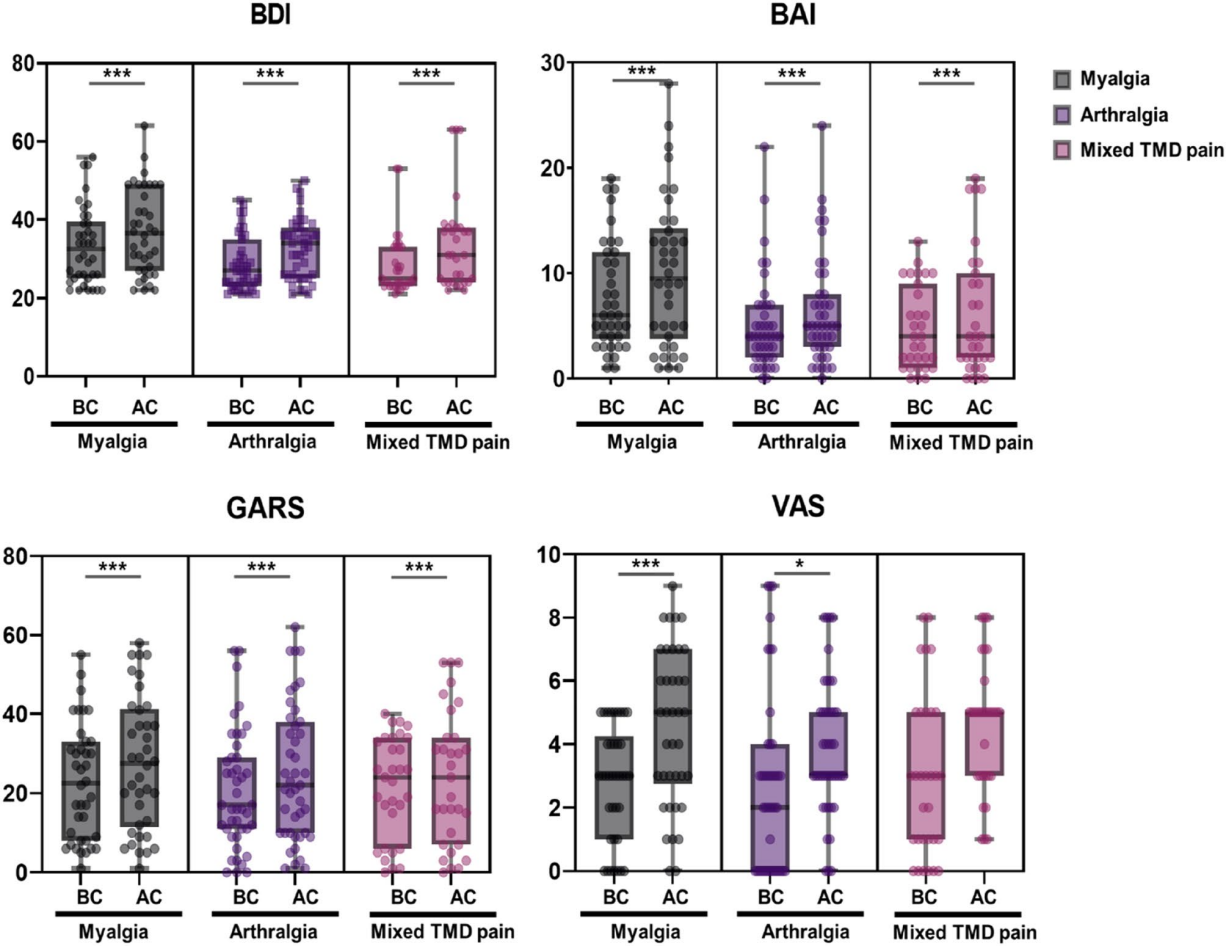
Comparison of Beck Depression Inventory (BDI), Beck Anxiety Inventory (BAI), Global Assessment of Recent Stress (GARS), and Visual Analog Scale (VAS) scores according to TMD pain subgroups. Results were obtained using a paired t-test. Statistical significance was set at p < 0.05. *p < 0.05, ***p < 0.001. TMD, temporomandibular disorder; BDI, Beck Depression Inventory; BAI, Beck Anxiety Inventory; GARS, Global Assessment of Recent Stress; VAS, visual analog scale; BC: before COVID-19, AC: after COVID-19, BC, total score in BC; AC, total score in AC.

When the data were divided into three subgroups and analyzed, the BDI and BAI scores differed significantly according to the origin of TMD pain (Fig. 2). Specifically, BDI-BC, BAI-BC, and BAI-AC were significantly higher in the myalgia group than in the arthralgia and mixed TMD pain groups. There were no significant differences between GARS-BC and GARS-AC among the three TMD subgroups.

Regarding BDI and BAI according to level, mild and moderate depression was observed in 36.84% and 63.16% of patients in the myalgia group. Moderate depression was significantly more frequent in the myalgia group than in the arthralgia (39.53%) and mixed TMD pain (35.48%) groups (p = 0.0371). Normal and severe depression was not observed in any TMD subgroup (Table 2).

**Table 2:** Comparison of discontinuous parameters before and after COVID-19.

Discontinuous parameters		Myalgia (n = 38)		Arthralgia (n = 43)		Mixed TMD pain (n = 31)		p-value
		n	(%)	n	(%)	n	(%)	
Sex	Female	30	(78.9%)	26	(60.5%)	18	(58.1%)	0.116^†^
	Male	8	(21.1%)	17	(39.5%)	13	(41.9%)	
BDI level_BC	1-Mild	**14**	**(36.84%)**	**26**	**(60.47%)**	**20**	**(64.52%)**	0.0371*^,^^**†**^
	2-Moderate	**24**	**(63.16%)**	**17**	**(39.53%)**	**11**	**(35.48%)**	
BDI level_AC	1-Mild	11	(28.95%)	15	(34.88%)	13	(41.94%)	0.5302^†^
	2-Moderate	27	(71.05%)	28	(65.12%)	18	(58.06%)	
BAI level_BC	0-Normal	24	(63.16%)	36	(83.72%)	22	(70.97%)	0.080^‡^
	1-Mild	10	(26.32%)	5	(11.63%)	9	(29.03%)	
	2-Moderate	4	(10.53%)	2	(4.65%)	0	(0%)	
BAI level_AC	0-Normal	16	(42.11%)	31	(72.09%)	21	(67.74%)	0.084^‡^
	1-Mild	15	(39.47%)	9	(20.93%)	6	(19.35%)	
	2-Moderate	6	(15.79%)	3	(6.98%)	4	(12.9%)	
	3-Severe	1	(2.63%)	0	(0%)	0	(0%)	
**Clinical and contributing factors**								
TMJ noise	No	7	(18.42%)	14	(32.56%)	11	(35.48%)	0.226^†^
	Yes	31	(81.58%)	29	(67.44%)	20	(64.52%)	
MOL	No	24	(63.16%)	31	(72.09%)	19	(61.29%)	0.561^†^
	Yes	14	(36.84%)	12	(27.91%)	12	(38.71%)	
Stiffness	No	19	(50.0%)	25	(58.14%)	15	(48.39%)	0.653^†^
	Yes	19	(50.0%)	18	(41.86%)	16	(51.61%)	
Locking	No	22	(57.89%)	29	(67.44%)	16	(51.61%)	0.374^†^
	Yes	16	(42.11%)	14	(32.56%)	15	(48.39%)	
Tinnitus	No	26	(68.42%)	34	(79.07%)	21	(67.74%)	0.451^†^
	Yes	12	(31.58%)	9	(20.93%)	10	(32.26%)	
Headache	No	**17**	**(44.74%)**	**37**	**(86.05%)**	**20**	**(64.52%)**	0.0005***^,^^**†**^
	Yes	**21**	**(55.26%)**	**6**	**(13.95%)**	**11**	**(35.48%)**	
Sleep problems	No	24	(63.16%)	34	(79.07%)	23	(74.19%)	0.269^†^
	Yes	14	(36.84%)	9	(20.93%)	8	(25.81%)	
Psychological stress	No	13	(34.21%)	23	(53.49%)	16	(51.61%)	0.176^†^
	Yes	25	(65.79%)	20	(46.51%)	15	(48.39%)	
Family hx.	No	33	(86.84%)	40	(93.02%)	26	(83.87%)	0.396^‡^
	Yes	5	(13.16%)	3	(6.98%)	5	(16.13%)	
Macrotrauma hx.	No	34	(89.47%)	39	(90.7%)	30	(96.77%)	0.567^‡^
	Yes	4	(10.53%)	4	(9.3%)	1	(3.23%)	
Bruxism	No	32	(84.21%)	28	(65.12%)	26	(83.87%)	0.070^†^
	Yes	6	(15.79%)	15	(34.88%)	5	(16.13%)	
Clenching	No	26	(68.42%)	23	(54.76%)	20	(64.52%)	0.431^†^
	Yes	12	(31.58%)	19	(45.24%)	11	(35.48%)	

TMD: temporomandibular disorder, MOL: mouth-opening limitation, BDI: Beck Depression Inventory, BAI: Beck Anxiety Inventory, BC: before COVID-19, AC: after COVID-19. level_BC: level in BC; level_AC: level in AC.

^†^Chi-square test, ‡Fisher’s exact test; p-value < 0.05 was considered significant. *p-value < 0.05, ***p-value < 0.001.

Significant results were bolded.

### Correlations among the psychological parameters

**Figure 3:**
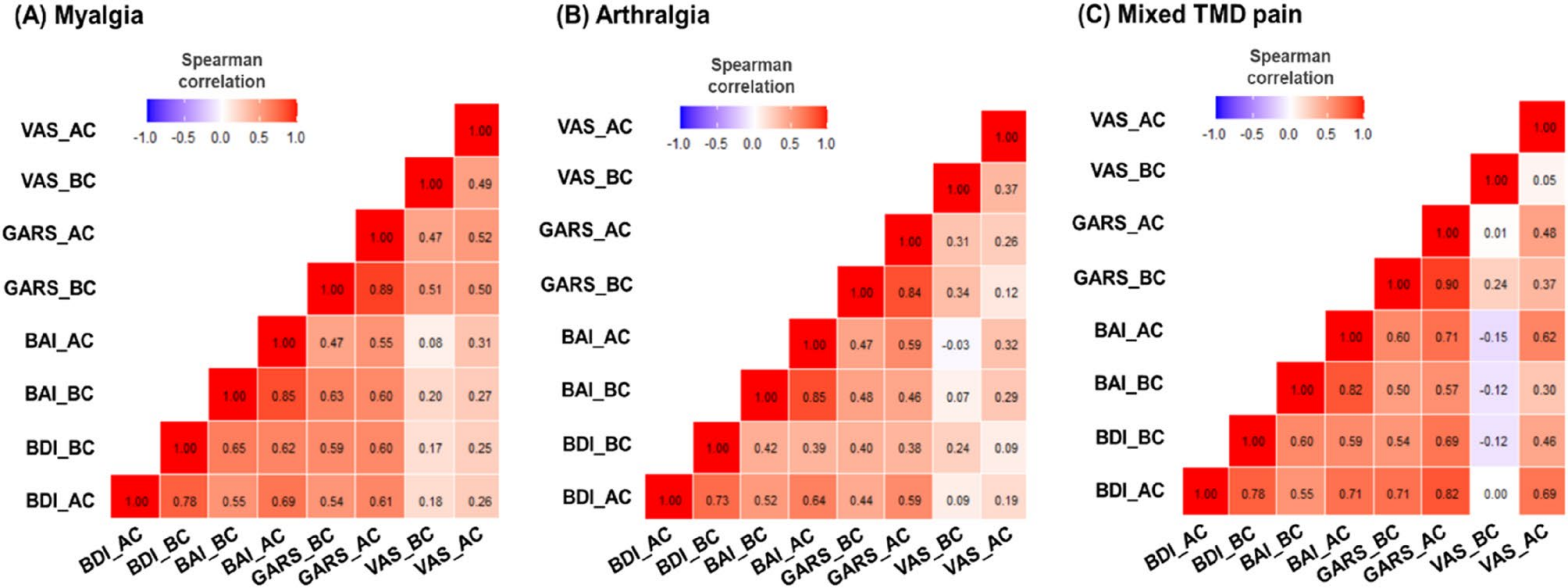
Correlation of Beck Depression Inventory (BDI), Beck Anxiety Inventory (BAI), Global Assessment of Recent Stress (GARS), and Visual Analog Scale (VAS) in TMD pain subgroups. Results were obtained via Spearman’s correlation analysis. TMD, temporomandibular disorder; BDI, Beck Depression Inventory; BAI, Beck Anxiety Inventory; GARS, Global Assessment of Recent Stress; VAS, visual analog scale; BC: before COVID-19, AC: after COVID-19, BC, total score in BC; AC, total score in AC.

Psychological distress-related factors (BDI, BAI, and GARS) showed a high positive correlation with each other, and the AC and BC scores of each factor were significantly positively correlated (Fig. 3).

In the myalgia group, BDI-AC was positively correlated with BDI-BC (r = 0.780), BAI-BC (r = 0.549), BAI-AC (r = 0.689), GARS-BC (r = 0.542), and GARS-AC (r = 0.614) (all p < 0.001). The depression, anxiety, and stress levels were significantly positively correlated with each other, and the AC and BC scores of each factor showed a high correlation. No significant correlation was observed between depression and the VAS scores for myalgia. However, there was a significantly positive correlation between GARS-BC and VAS-BC (r = 0.498, p < 0.01), and between GARS-AC and VAS-AC (r = 0.523, p < 0.01).

In the arthralgia group, factors positively correlated with BDI-AC were BDI-BC (r = 0.730), BAI-BC (r = 0.518), BAI-AC (r = 0.636), GARS-BC (r = 0.442), and GARS-AC (r = 0.590) (all p < 0.001). BDI-AC was not significantly correlated with VAS-BC and VAS-AC, though GARS-BC was positively correlated with VAS-BC (r = 0.309, p = 0.043). In the mixed TMD pain group, factors positively correlated with BDI-AC were BDI-BC (r = 0.779, p < 0.001), BAI-BC (r = 0.552, p < 0.01), BAI-AC (r = 0.710, p < 0.001), GARS-BC (r = 0.711, p < 0.001), and GARS-AC (r = 0.820, p < 0.001). In the mixed TMD group, BDI-AC was significantly positively correlated with VAS-AC (r = 0.689, p < 0.001).

### Clinical factors

Among the clinical factors, TMJ sounds (n = 80, 71.4%) were most frequently observed, followed by muscle stiffness (n = 53, 47.3%), jaw locking (n = 45, 40.2%), and mouth-opening limitation (n = 38, 33.9%). The proportions of all clinical factors did not differ significantly among the three TMD subgroups.

### Contributing factors

The most frequent contributing factor was psychological distress (n = 60, 53.6%), followed by clenching (n = 42, 37.5%), headache (n = 38, 33.93%), tinnitus (n = 31, 27.7%), sleep problems (n = 31, 27.7%), bruxism (n = 26, 23.2%), family history (n = 13, 11.6%), and microtrauma history (n = 9, 8.04%). Among the eight contributing factors, only prevalence of headache differed among the TMD subgroups. Headache was most common in the myalgia group (n = 21, 55.26%), followed by the mixed TMD pain (n = 6, 35.48%), and arthralgia groups (n = 11, 13.95%) (p < 0.001). The remaining factors were not significantly different among the TMD subgroups.

### Results of the generalized linear model of factors that increase depression score

Table 3 presents the parameters related to the increase in depression score during COVID-19. Using generalized linear models, we analyzed predictors that increased BDI-AC (total score of BDI during COVID-19). The TMD subgroup did not show an increase in the total BDI-AC score. A decrease in age (β = − 0.09; 95% confidence interval [CI] − 0.17 to − 0.01) and increase in symptom duration (β = 0.02; 95% CI 0.01–0.03) and VAS-AC (β = 1.09; 95% CI 0.47–1.70) were significant predictors of an increase in the total BDI score. As investigated in the correlation, psychological distress-related factors were related to the increase in BDI_AC. That is, BAI mild level-BC (β = 6.07; 95% CI 2.12–10.02), BAI mild level-AC (β = 3.95; 95% CI 0.99–6.91), BAI moderate level-AC (β = 13.52; 95% CI 9.26–17.79), BAI severe level-AC (β = 21.19; 95% CI 8.46–33.93), BAI-BC (β = 0.54; 95% CI 0.19–0.89), BAI-AC (β = 0.79; 95% CI 0.57–1.01), GARS-BC (β = 0.18; 95% CI 0.07–0.29), and GARS-AC (β = 0.26; 95% CI 0.18–0.35) were related to the increase in the total BDI score. Among the clinical and contributing factors, muscle stiffness (β = 5.06; 95% CI 2.40–7.72) and psychological stress (β = 3.15; 95% CI 0.34–5.95) were significant parameters for increase in BDI-AC.

**Table 3:** Parameters related to the increase of depression after COVID-19.

Parameters	Independent	BDI_AC^†^					BDI level 3_AC compared to BDI level 2_AC^‡^			
		β estimate	95% CI		p-value		OR	95% CI		p-value
**Epidemiology and clinical characteristics**										
Age (year)		**− 0.09**	**− 0.17**	**− 0.01**	0.037*		0.98	0.95	1.01	0.129
Sex [ref = male]	Female	− 1.98	− 6	2.05	0.332		**2.08**	**1.33**	**2.66**	0.008**
Symptom duration (day)		**0.02**	**0.01**	**0.03**	0.0012**		1	1	1	0.982
Group [ref = myalgia]	Arthralgia	− 0.3	− 3.74	3.14	0.863		2.17	0.56	8.49	0.264
	Mixed TMD pain	0.64	− 3.08	4.36	0.730		1.61	0.38	6.77	0.518
VAS-BC		− 0.03	− 0.62	0.56	0.917		1.1	0.9	1.36	0.355
VAS-AC		**1.09**	**0.47**	**1.7**	0.0007***		**1.32**	**1.02**	**1.71**	0.034*
**Psychological distress**										
BAI level-BC [ref = 0 (normal)]	1-mild	**6.07**	**2.12**	**10.02**	0.003**		1.33	0.17	10.6	0.789
	2-moderate	5.99	− 0.4	12.38	0.066		0.45	0.01	14.85	0.654
BAI level-AC [ref = 0 (normal)]	1-mild	**3.95**	**0.99**	**6.91**	0.009**		3.28	0.99	10.9	0.053
	2-moderate	**13.52**	**9.26**	**17.79**	< 0.0001***		> 999.99	< 0.001	> 999.99	0.963
	3-severe	**21.19**	**8.46**	**33.93**	0.001**		> 999.99	< 0.001	> 999.99	0.999
BAI-BC		**0.54**	**0.19**	**0.89**	0.003**		1.05	0.9	1.24	0.525
BAI-AC		**0.79**	**0.57**	**1.01**	< 0.0001***		**1.21**	**1.06**	**1.39**	0.005**
GARS-BC		**0.18**	**0.07**	**0.29**	0.001**		1.02	0.98	1.07	0.326
GARS-AC		**0.26**	**0.18**	**0.35**	< 0.0001***		**1.05**	**1.01**	**1.09**	0.023*
**Clinical and contributing factors**										
TMJ noise [ref = none]	Presence	− 0.04	− 3.16	3.09	0.981		**0.31**	**0.1**	**1**	0.049*
MOL [ref = none]	Presence	− 2.94	− 5.91	0.02	0.052		0.93	0.31	2.82	0.904
Muscle stiffness [ref = none]	Presence	**5.06**	**2.4**	**7.72**	< 0.0001***		**3.02**	**1.05**	**8.68**	0.041*
Locking [ref = none]	Presence	0.4	− 2.48	3.28	0.7827		0.62	0.21	1.82	0.386
Tinnitus [ref = none]	Presence	**4.1**	**1.03**	**7.18**	0.009**		1.54	0.48	5.01	0.469
Headache [ref = none]	Presence	**4**	**1.1**	**6.9**	0.007**		1.39	0.44	4.36	0.578
Sleep problem [ref = none]	Presence	− 0.09	− 3.24	3.06	0.9554		1.54	0.48	5.01	0.470
Psychological stress [ref = none]	Presence	**3.15**	**0.34**	**5.95**	0.028*		**4.5**	**1.51**	**13.45**	0.007**
Family hx [ref = none]	Presence	0.39	− 4.04	4.83	0.861		0.28	0.05	1.74	0.173
Macrorauma hx [ref = none]	Presence	2.08	− 3.09	7.26	0.426		2.92	0.46	18.64	0.258
Bruxism [ref = none]	Presence	− 0.34	− 3.68	3.01	0.810		1.21	0.36	4.08	0.762
Clenching [ref = none]	Presence	1.55	− 1.36	4.47	0.292		**7.09**	**2.23**	**22.55**	0.0009***

^†^Generalized linear model adjusted by BDI-BC, ^‡^Multiple logistic regression analysis adjusted by BDI level-BC, significant p-value set at < 0.05, and significant results were bolded. *p-value < 0.05, **p-value < 0.01, ***p-value < 0.001.

TMD: temporomandibular disorder; MOL: mouth-opening limitation; BDI: Beck Depression Inventory; BAI: Beck Anxiety Inventory; GARS: Global Assessment of Recent Stress; BC: before COVID-19, AC: after COVID-19, OR: odds ratio; CI: confidence interval; ref: reference; -BC: total score in BC; -AC: total score in AC; level-BC: level in BC; level-AC: level in AC.

### Results of multiple logistic regression analysis of factors that increase depression level

In the multiple regression analysis predicting BDI level 3-AC compared to BDI level 2-AC, several significant predictors were derived. The TMD subgroup did not increase the BDI-AC level. Among epidemiological factors, female sex (odds ratio [OR] = 2.08; 95% CI 1.33–2.66) was a significant predictor of an increase in the BDI-AC level. Further, BAI-AC (OR = 1.21, 95% CI 1.06–1.39) was significantly related with psychological distress-related factors. Among the clinical and contributing factors, clenching habit (OR = 7.09, 95% CI 2.23–22.25), psychological distress (OR = 4.50, 95% CI 1.51–13.45), muscle stiffness (OR = 3.02, 95% CI 1.05–8.68), and TMJ sounds (OR = 0.31, 95% CI 0.10–1.00) were associated with an increase in BDI levels from moderate to severe. Bruxism was not correlated with an increase in the depression level; however, clenching was a major predictor of a > sevenfold increase in the BDI level.

## Discussion

Our findings appear to support the hypothesis that the COVID-19 pandemic lockdown influenced depression in painful TMD, albeit with individual responses. Our study also investigated factors influencing the increase in depression levels in patients with TMD in the context of COVID-19 using multiple regression analysis. In the analysis adjusted for BDI level-BC, psychological stress (OR = 4.5), clenching habit (OR = 7.09), female sex (OR = 2.08), muscle stiffness (OR = 3.02), and TMJ sounds (OR = 0.31) were significant predictors of increased BDI levels in patients with painful TMD. The COVID-19 lockdown is reportedly a major stressful life event globally since the 2020 WHO pandemic declaration, and this unprecedented pandemic has been associated with pain aggravation^2^. Depression is a major psychological issue, and the co-occurrence of depression and body pain is commonly encountered globally. Additionally, depression and TMD pain can be bidirectional; compared to non-depressed patients, those with moderate-to-severe depressive symptoms are almost equally likely to develop TMD (OR, 1.2–1.6)^29^. Furthermore, depression exacerbates TMD pain, which negatively affects depression and constitutes substantial economic, social, and personal costs^30^.

In this study, there was a strong positive correlation between depression, anxiety, and psychological distress in patients with TMD during COVID-19. Furthermore, the BDI, BAI, GARS, and VAS scores were significantly increased during the COVID-19 pandemic compared to those during the pre-COVID-19 duration. Increased depression is associated with increased anxiety, and the symptoms of psychological distress are predictors of poor outcomes in treatment of painful disease^31^. A positive correlation between depression and anxiety symptoms and stress has been reported^32,33^. This close relationship has long been recognized as a hidden mental health network^34^. Although substantial work is required to increase awareness of depression, anxiety, and stress in patients with painful TMD during COVID-19, the last decade has witnessed enormous progress in both the recognition and management of general body pain and psychological distress.

The underlying mechanism by which painful TMD and depression are linked during COVID-19 is more of a suggestion than a certainty. Anxiety and depression are common in patients with body pain and usually account for neuroplastic changes in the central nervous system^35^. Depression may occur as a result of the decreased availability of monoamine neurotransmitters such as 5-hydroxytryptamine and norepinephrine in the central nervous system^36^. Monoamine neurotransmitters are also important in the occurrence and development of pain. Furthermore, glutamate and its receptor subtypes (N-methyl-D-aspartic acid and α-amino-3-hydroxy-5-methyl-4-isoxazolepropionic acid receptors), have been found to be involved in the occurrence and development of chronic pain and depression^37^. Additionally, the surrounding inflammatory response causes pain and depression; thus, inflammatory response-mediated pain may be more strongly associated with depression^38^. Inflammatory signals can induce changes in neurotransmitter metabolism, neuroendocrine functions, and neuroplasticity. Patients with decreased function in the prefrontal cortex, hippocampus, and other depression-related structures also have decreased brain-derived neurotrophic factor expression^39^. Further research is needed to determine how the COVID-19 pandemic has affected these neurophysiological changes in patients with painful TMD.

In this study, both muscle stiffness and TMJ sounds were predictors of increased depression. TMJ sounds such as clicking or crepitation are among the most common symptoms. Since TMJ sounds are not always considered a problem, but rather a risk factor, TMJ clicking may be a normal variant rather than a disorder^40^. However, although TMJ sounds did not cause any particular pain or functional limitation, it was significantly associated with an increase in depression; therefore, clinicians should not overlook TMJ sounds. The presence of a clenching habit was a predictor of a 2.23-fold increase in depression. Parafunctional activities are usually harmless until the forces exerted exceed structural tolerance^41^. Parafunctional habits, such as bruxism, tooth clenching, gum chewing, biting foreign objects, and prolonged nail biting, might increase the risk of developing TMD^42^. In particular, bruxism and clenching reportedly lead to joint space reduction, followed by disc compression and pain in the masticatory muscles^40^. Additionally, psychological distress has been closely linked to clenching and bruxism. Winocur et al. conducted a study on the association of self-reported bruxism with perceived stress^43^. Further, in a study performed by Abekura et al. in which stress was assessed by measuring salivary chromogranin A levels, the findings suggested a relationship between psychological stress and nocturnal bruxism^44^. However, this requires further investigation. According to Smardz et al., the intensity of nocturnal bruxism is not significantly correlated with self-reported perceived stress and depression^45^. Additionally, Ohlmann et al. aimed to identify associations between definite nocturnal bruxism, chronic stress, and sleep quality, and reported that chronic stress and sleep quality do not seem to be associated with nocturnal bruxism^46^.

Female sex was an important predictor of a 2.08-fold increase in depression in patients with painful TMD. That is, females with painful TMD experienced more psychological depression than males during the COVID-19 pandemic. In general, TMD is predominant in women^47^. According to Birgitta Häggman-Henrikson et al., women are 2.37 times more likely to develop orofacial pain than men and are more likely to experience chronic orofacial pain^48^. A previous meta-analysis reported sex differences in depression, and the prevalence of women experiencing major depression was twice that of men, indicating a major health disparity^49^. Women are more vulnerable than men to social and economic stresses such as the COVID-19 pandemic^50^. Thus, the burden of depression falls disproportionately on women with painful TMD during COVID-19. Among epidemiological factors, younger age is associated with increased depression. A recent study showed that younger individuals were more vulnerable to stress, anxiety, and depression during COVID-19 than older individuals^51^. During a persisting pandemic, age- and sex-specific interventions may be necessary to control pain and psychological distress in patients with painful TMD.

Furthermore, a longer symptom duration was a significant predictor for higher total BDI scores in patients with painful TMD. The causes of TMD are complex, difficult to resolve, and prone to becoming chronic. Chronic TMD pain must be considered in the biopsychosocial model, which considers TMD symptoms to be the result of a complex and dynamic interaction among biological, psychological, and social factors^52^. The underlying mechanisms include genetic factors, previous pain experiences, and traumatic events that could be physical or emotional. Central hypersensitivity symptoms in chronic pain are associated with stronger emotional distress in patients with TMD^53^. Additionally, patients with TMD accompanied with depression and anxiety have an increased risk of joint and muscle pain, respectively^54^. Patients with chronic musculoskeletal pain have higher depression levels than those without pain^55^.

Chronic pain may be triggered by psychosocial stressors or physical/biological factors, which may occur preferentially in individuals with a vulnerable stress response system. The COVID-19 pandemic has several characteristics that could potentially increase the prevalence of chronic pain, and it should be noted that the stressors could progress over months.

For the first time, in this study, we comprehensively investigated the factors affecting depression in patients with painful TMD during the COVID-19 pandemic. In patients with painful TMD, depression levels increased during the COVID-19 pandemic, and psycho-emotional status such as anxiety and distress and their pain intensity were also negatively affected. We investigated the influence of several clinical factors on depression in patients. Nonetheless, our study had several limitations. First, the cross-sectional study design has limitations in accurately reflecting the actual epidemiology and clarifying the relationship between parameters. Second, no control group was included. Further research is needed to determine how painful TMD is affected by the COVID-19 situation by comparing patients with TMD and healthy controls who do not have painful TMD. Finally, there might have been a patient memory bias since the questionnaire was not implemented before the WHO declaration of pandemic; rather, the patients answered the questionnaire based on recalled responses. However, it is worth noting that TMD was systematically diagnosed by TMD specialists according to the DC/TMD criteria, and a psychological status analysis during the COVID-19 pandemic was performed.

## Data Availability

The datasets generated and analyzed during the current study are not publicly available due to the nature of clinical data, including patient personal information, but are available upon reasonable request from the corresponding author after discussion with the KHU-IRB.
